# Flight State Identification of a Self-Sensing Wing via an Improved Feature Selection Method and Machine Learning Approaches

**DOI:** 10.3390/s18051379

**Published:** 2018-04-29

**Authors:** Xi Chen, Fotis Kopsaftopoulos, Qi Wu, He Ren, Fu-Kuo Chang

**Affiliations:** 1 Shanghai Engineering Research Center of Civil Aircraft Health Monitoring, Shanghai Aircraft Customer Service Co., Ltd., Shanghai 200241, China; renhe@comac.cc; 2 Department of Mechanical, Aerospace, and Nuclear Engineering, Rensselaer Polytechnic Institute, Troy, NY 12180, USA; kopsaf@rpi.edu; 3 School of Electronic, Information and Electrical Engineering, Shanghai Jiao Tong University, Shanghai 200240, China; wuqi7812@sjtu.edu.cn; 4 Department of Aeronautics and Astronautics, Stanford University, Stanford, CA 94305, USA; fkchang@stanford.edu

**Keywords:** self-sensing wing, feature extraction, feature selection, flight state identification, machine learning

## Abstract

In this work, a data-driven approach for identifying the flight state of a self-sensing wing structure with an embedded multi-functional sensing network is proposed. The flight state is characterized by the structural vibration signals recorded from a series of wind tunnel experiments under varying angles of attack and airspeeds. A large feature pool is created by extracting potential features from the signals covering the time domain, the frequency domain as well as the information domain. Special emphasis is given to feature selection in which a novel filter method is developed based on the combination of a modified distance evaluation algorithm and a variance inflation factor. Machine learning algorithms are then employed to establish the mapping relationship from the feature space to the practical state space. Results from two case studies demonstrate the high identification accuracy and the effectiveness of the model complexity reduction via the proposed method, thus providing new perspectives of self-awareness towards the next generation of intelligent air vehicles.

## 1. Introduction

The current state sensing and awareness of flight vehicles relies on traditional sensors and detection devices mounted on different locations of the vehicle, e.g., Pitot tubes installed in front of the nose for airspeed measurement, transducers located on each side of the fuselage for angle of attack detection. Inspired by the unsurpassed flight capabilities of birds, a novel “fly-by-feel” (FBF) concept has been recently proposed for the development of the next generation of intelligent air vehicles that can “feel”, “think”, and “react” [1,2]. Such bio-inspired systems will not only be able to sense the environment (temperature, pressure, aerodynamic forces, etc.), but also be able to think in real-time and be aware of their current flight state and structural health condition. Further, such systems will react intelligently under various situations and achieve superior performance and agility. Compared with the traditional approaches, this FBF concept has the following advantages: (1) structural complexity reduction by integrated structures with self-sensing ability, (2) structural health on-line monitoring through embedded multi-functional materials, (3) autonomous flight control and decision-making based on self-awareness [2]. Towards this end, great challenges have been posed to the current structural design and data processing methods with a departure from the existing technologies.

**Figure 1:**
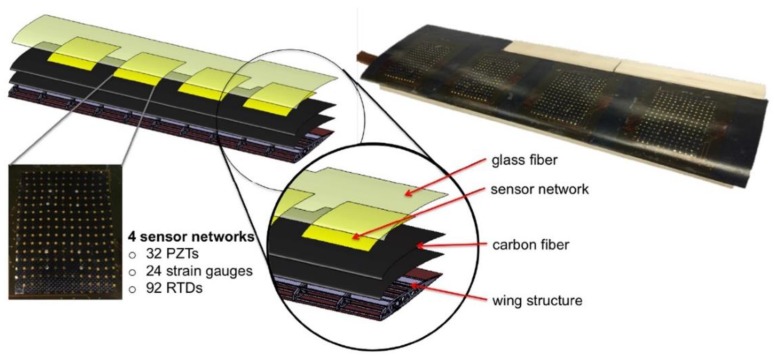
The self-sensing composite wing design [2].

Recent years have seen the development of different sensing network architectures and simulations [3,4,5,6], among which, an expandable network made of polymer-based substrates was designed by the Structure and Composites Lab (SACL) at Stanford University. This network contains many micro-nodes which have the potential to integrate micro-sensors, actuators and electronics for different applications [7]. Based on the development of integration and fabrication techniques [8,9,10], a smart structure with the sensor network monolithically embedded in the layup of a composite UAV wing was successfully fabricated [11]. This smart wing consists of four sensor networks and each network is integrated with strain gauges, resistive temperature detectors (RTD) and piezoelectric lead zirconate titanate (PZT) transducers. Specifically, the strain gauge is used to measure the wing strain distribution and identify any potentially dangerous areas. RTD detects the temperature distribution in order to provide the temperature compensation [12]. PZT transducers can be used for both active and passive measurements. In the active mode, they can be used for damage detection and structural health monitoring while in passive mode, the wing structural vibration during flying can be captured to reflect the air dynamic characteristics [11]. The wing configuration is shown in Figure 1.

After realizing sensing ability through multi-functional structures development, the next step is to equip the smart wing with thinking and judging capability, i.e., the structure is expected to be aware of surroundings and identify its current flying state. There have been studies devoted to addressing the related identification problem based on either strain or vibration signals obtained from experiments. Huang et al. studied the active flutter control and closed-loop flutter identification and a fast-recursive subspace method was applied in high-dimensional aero-servo-elastic system. The wind tunnel test showed that the natural frequency and modal damping ratios of the flutter modes can be precisely tracked [13]. Pang and Cesnik employed non-linear least squares fit and Kalman filtering to obtain wing shape information and rigid body attitude. Results revealed that the Kalman filter has good performance in the presence of sensor noise [14]. For elastic deformation, Sodja et al. conducted a dynamic aeroelastic wind tunnel experiment under harmonic pitching excitations, experimental data including the bending and torsion deformation were consistent with the elastic analysis model developed by the Delft University of Technology [15]. For more general flight states, Kopsaftopoulos and Chang established a stochastic global identification method using PZT signals from both time and frequency domain based on developed Vector-dependent Functionally Pooled (VFP) model [2,16,17]. A large range of airspeeds and angles of attack were considered in the VFP-based identification framework and the structural dynamics of the composite wing could be captured and predicted. 

Overall, the above data processing approaches mainly belong to state space methods and improved time series analysis. Based on the previous study yet from another perspective, if we can extract distinguished features from the continuous coupled structural aerodynamic behavior, it is possible to identify the flight state directly using the limited features instead of detailed characterization of the structural responses. Machine learning techniques can be employed to establish the mapping relationship from the feature space to the practical state space. 

Facing a series of signals generated from the embedded sensor network, one of the main challenges is what kind of features should be extracted and whether these features are useful for classification. A set of features without careful selection and evaluation may lead to poor results whatever superior machine learning models are applied. Feature engineering is such a research field including feature extraction and selection. For a period of time series signals with noise, various statistical features can be calculated such as the mean value, standard deviation, peak value, kurtosis, etc. from both time domain and frequency domain [18], a feature pool is then created with different number of features depending on the characteristics of the signals [19,20,21]. More features are encouraged to avoid missing important candidates with superior classification performance. The next step is feature selection in which a limited subset is obtained by eliminating less effective features. It reduces model dimension and computational time [22]. Generally, feature selection can be divided into three categories as filter, wrapper and embedded. Filter methods rank the variables completely separate to the model used for classification. The assignment of feature importance is based on information generated by some statistical algorithms. Filter methods are computationally simple and fast without the interaction with the classifier and feature dependencies [23]. Embedded solutions select salient features as part of the learning process of the model, which can be linear regression, support vector machine, decision tree, random forest, etc. These methods integrate the subset selection into the model construction but are difficult to adjust for the optimal search [24]. The third category is wrapper, in which features are selected based on the performance of a given model by searching the possible subsets space and assessing the performance of the given model on each subset, models can be various learning machines [25]. Although wrapper methods often achieve sound classification performance by considering the feature dependencies, the frequent interactions between feature subset search and the classifier cause high computational costs [26]. 

**Figure 2:**
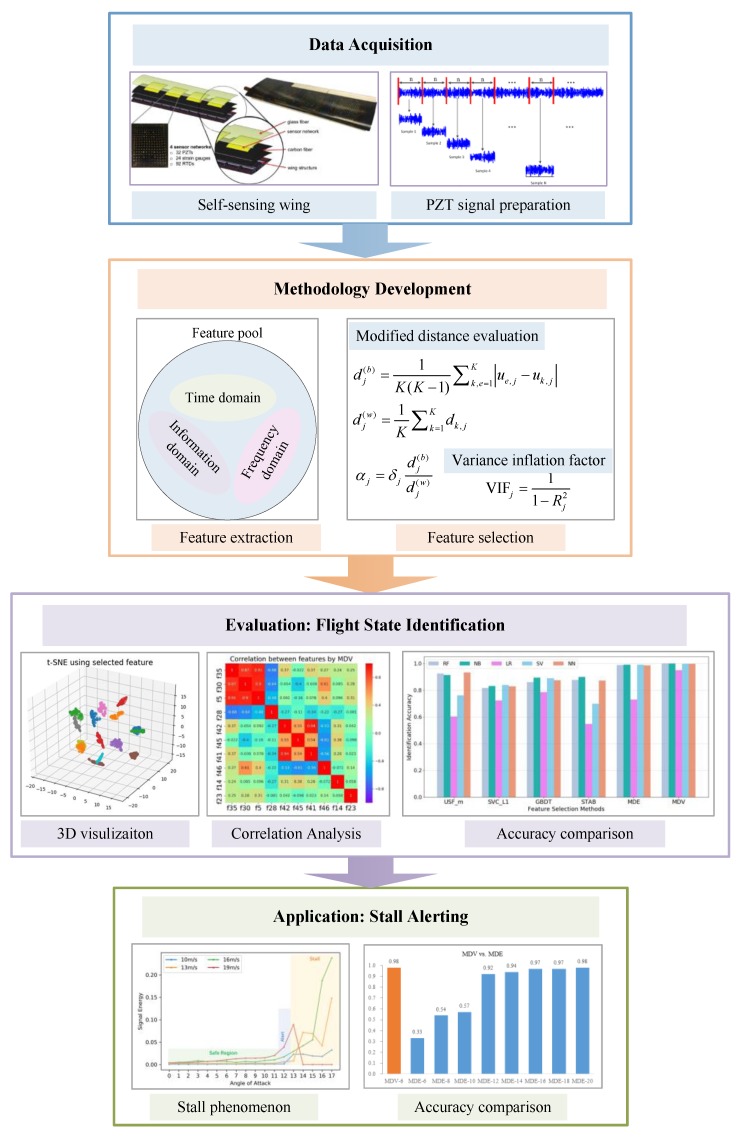
Framework of the proposed methodology.

We have demonstrated the effectiveness of establishing the mapping relationship from the feature space to the flight state space through neural networks modelling [27]. This paper significantly improves the previous work by creating a much larger feature pool and considering the co-linearity among various features. To sum up, the objective of this paper is the introduction and evaluation of a novel feature selection method for accurate flight state identification of a self-sensing wing structure based on experimental vibration data recorded by piezoelectric sensors under multiple flight states. The developed method belongs to the filter family and is capable of obtaining a group of most important features for classification with low mutual dependency. The framework of the data acquisition, methodology development, evaluation and application is shown in Figure 2.

The rest of the paper is organized as follows: Section 2 presents the problem statement. Section 3 focuses on the feature extraction and feature selection in which the novel filter algorithm is introduced. Two case studies including the general flight state identification and the stall detection and alerting are conducted in Section 4 followed by their results and discussions in Section 5. Concluding marks are made in the last section.

## 2. Problem Statement

The problem statement of this work is as follows: based on signals collected from the PZT sensors embedded in the self-sensing wing through a series of experiments under varying flight states, develop a feature selection method that is capable of obtaining limited useful features for flight state identification with high accuracy and low model complexity. Specifically, the coupled aerodynamic-mechanical responses represent different flight states, with each state characterized by a specific angle of attack (AoA) and airspeed and kept constant during the data collection. The first problem is that whether a few salient features can be extracted from a period of vibrational time series (e.g., thousands of data points) as a representation of the corresponding flight state. In this way, we can skip the investigation into the detailed aeroelastic behavior and use the limited features to identify the specific flight state directly instead of using the entire lengthy signal. This would significantly reduce the complexity of the flight state characterization. The second problem is how to guarantee the effectiveness of selected features. If the selected strong features are highly correlated with each other, they will exhibit similar identification ability which are still away from the optimal subset. 

The above two problems constitute the motivation of this study and are addressed in the following approaches: firstly, a large number of features is extracted to cover a wide range of descriptions of the flight state. Then, a modified distance evaluation algorithm is conducted to obtain a subset of individually powerful features followed by the combination of a variance inflation factor algorithm to reduce high dependency among features in the subset. Machine learning models are employed to evaluate the above method for multiple flight states identification as well as a specific case of stall detection and alerting. 

The main novel aspects of this study include:

(1) A large feature pool is created covering up to 47 different features from the time, frequency and information domains.
(2) A novel filter feature selection method is developed by combining a modified distance evaluation algorithm and a variance inflation factor.
(3) The flight state identification is treated as a classification problem by establishing the mapping relationship from the feature space to the physical space characterized by varying angle of attack and airspeed of the self-sensing wing structure in wind tunnel experiments.
(4) The application on stall detection and alerting with high identification accuracy provides new perspectives for autonomous flight control with real-time flight state monitoring.

## 3. Methodology Development

In this section, a novel filter feature selection method is proposed via the combination of a modified distance evaluation algorithm and a variance inflation factor. In order to obtain sufficient feature candidates, a large feature pool is firstly created by extracting features covering a wide range. The output of this method is a feature subset consisting of most salient features with low correlation, which is able to represent a lengthy time-series signal of the wing structural response under certain flight state.

### 3.1. Feature Extraction

Feature extraction relies heavily on experts’ knowledge, it is encouraged to extract different kinds of features, as many as possible in case of missing useful ones. In this study, we intend to create a large feature pool from three main sources, namely the time, frequency and information domains. 

In time domain, 25 statistical features are calculated including 12 commonly used features such as mean, standard deviation, variance, peak, mean absolute deviation, etc. and 13 un-dimensional features such as crest factor, shape factor and a series of normalized central moments. The expressions of all time domain features are listed in Table 1. In terms of their physical insights, *t*_1_–*t*_12_ may reflect the vibration amplitude and energy while *t*_13_–*t*_25_ may represent the series distribution of the signal in time domain.

Previous studies employed Fast Fourier Transform (FFT) to convert the time series into frequency spectrum [19,20]. However, the signal instances from the wind tunnel experiments are samples of a stochastic process with considerable noise. Welch’s method improves FFT by shortening the signals and averaging, and thus the peaks are smoothed for noise reduction [28]. Herein, a sample-long Hamming data window with 90% overlap is used for the Welch-based spectral estimation. A series of power spectrum *y*(*k*) without *log* transformation is then used for frequency domain feature extraction. Thirteen statistical features such as mean spectrum, spectrum center, root mean square spectrum, etc. and their mathematical expressions are shown in Table 2. *f*_1_ may indicate the vibration energy in the frequency domain. *f*_2–4_, *f*_6_, *f*_10–13_ may describe the convergence of the spectrum power. *f*_5_, *f*_7–9_ may show the position change of the main frequency.

In electroencephalograph (EEG) analysis for neural diseases diagnosis and vibration analysis for mechanical defects, fractal dimensions from computational geometry and entropies from information theory have demonstrated effectiveness in early diseases/fault diagnosis [29,30]. Inspired by that, a group of complex features are employed and their terminologies are Multi-Scale Entropy, Partial Mean of Multi-Scale Entropy, Petrosian Fractal Dimension, Higuchi Fractal Dimension, Fisher Information, Approximate Entropy, and Hurst Exponent, respectively.

Multi-Scale Entropy (MSE) introduces the scale factor based on the sample entropy to measure the complexity of signal under different scale factors [31]. It is calculated as:

(1) $$\mathrm{MSE}=\{\tau |SampEn(\tau ,m,r)=-\ln[C^{r,m+1}(r)/C^{r,m}(r)]\}$$

where τ is the scale factor, *m* is the embedding dimension and *r* is the threshold. Here *m =* 2, *r =* 0.2 * standard deviation, τ=12.

The first three values are selected due to the relatively high distinction among different classes. Also, an integrated non-linear index called Partial Mean of Multi-Scale Entropy (PMMSE) is used to simultaneously reflect the mean value and variation trend of MSE [32], which is expressed as:

(2) $$\mathrm{PMMSE}=(1+|\mathrm{Ske}|/3)\cdot MSE_{a}$$

where $\mathrm{Ske}=3(MSE_{a}-MSE_{b})/MSE_{c}$, *MSE_a_*, *MSE_b_*, *MSE_c_* represent mean, median and standard deviation of MSE(τ)=[MSE(1),MSE(2),…,MSE(12)].

Fractal dimension characterizes the space filling capacity of a pattern that changes with the scale at which it is measured [33]. Herein, two approaches are used as Petrosian Fractal Dimension (PFD) and Higuchi Fractal Dimension (HFD). PFD is calculated as:

(3) $$\mathrm{PFD}=\frac{\log_{10}N}{\log_{10}N+\log_{10}(N/(N+0.4N_{\delta }))}$$

where *N* is the length of the signal and $N_{\delta }$ is the number of sign changes in the signal derivative [30].

In terms of HFD, firstly *k* new series are constructed from the original signal $\left[x_{1},x_{2},\ldots ,x_{N}\right]$ by $\left[x_{m},x_{m+k},x_{m+2k},\ldots ,x_{m+\left\lfloor (N-m)/k\right\rfloor k}\right]$, where *m* = 1, 2, …, *k*. Secondly the length L(m,k) for each new series is calculated as:

(4) $$L(m,k)=\frac{\sum_{i=2}^{\left\lfloor (N-m)/k\right\rfloor }\left|x_{m+ik}-x_{m+(i-1)k}\right|(N-1)}{\left\lfloor (N-m)/k\right\rfloor k}$$

and the average length $L(k)=\left\lfloor \sum_{i=1}^{k}L(i,k)\right\rfloor /k$. After $k_{\max}$ repetitions, a least-squares method is used to obtain the best slope that fits the curve of ln(L(k)) versus ln(1/k), which is defined as the Higuchi Fractal Dimension. For details, please refer to [34].

Fisher Information (FI) measures the expected value of the observed information [35]. Its mathematical expression using normalized singular spectrum is:

(5) $$\mathrm{FI}=\sum_{i=1}^{M-1}\frac{{\left({\bar{\sigma }}_{i+1}-{\bar{\sigma }}_{i}\right)}^{2}}{{\bar{\sigma }}_{i}}$$

where ${\bar{\sigma }}_{i}$ is the normalized value through ${\bar{\sigma }}_{i}=\sigma_{i}/\sum_{j=1}^{M}\sigma_{j}$, and *M* is the number of singular value.

Approximate Entropy (ApEn) quantifies the amount of regularity and the unpredictability of fluctuations of a signal [36], which is computed in the following procedures:

(1) Set the input as $\left[x_{1},x_{2},\ldots ,x_{N}\right]$.
(2) Construct the subsequence $x(i,m)=\left[x_{i},x_{i+1},\ldots ,x_{i+m-1}\right]$ for 1≤i≤N−m, where *m* is the subsequence length.
(3) Construct a set of subsequences {x(j,m)}={x(j,m)|j∈[1,…N−m]}, where x(j,m) is defined in Step (2).
(4) For each x(i,m)∈{x(j,m)}, $C(i,m)=\frac{\sum_{j=1}^{N-m}k_{j}}{N-m}$, where $k_{j}=\{\begin{array}{cc} 1 & \mathrm{if}\;|x(i,m)-x(j,m)|<r\\ 0 & \mathrm{otherwise} \end{array}$.
(5) ApEn is calculated as:
  (6) $$\mathrm{ApEn}(m,r,N)=\frac{1}{N-M}\left[\sum_{i=1}^{N-m}\ln\frac{C(i,m)}{C(i,m+1)}\right]$$

Hurst Exponent (HST) measures the long-term memory of a signal. It is used to quantify the relative tendency of the signal either to regress to the mean or to cluster in a direction [37]. For time series $X=[x_{1},x_{2},\ldots ,x_{N}]$, its accumulated deviation within range *T* is calculated as $X(t,T)=\sum_{i=1}^{t}\left(x_{i}-\bar{x}\right)$, where $\bar{x}=\frac{1}{T}\sum_{i=1}^{T}x_{i}$, t∈[1,2,…,N]. Then:

(7) $$\frac{R(T)}{S(T)}=\frac{\max(X(t,T))-\min(X(t,T))}{\sqrt{(1/T)\sum_{t=1}^{T}{\left[x(t)-\bar{x}\right]}^{2}}}$$

The slope of ln(R(n)/S(n)) versus ln(n) for n∈[2,3,…,N] is defined as the Hurst Exponent.

In summary, abbreviations of the complex features extracted from information domain are listed in Table 3.

### 3.2. Feature Selection

Feature extraction guarantees a wide coverage of the object descriptions from various aspects while feature selection ensures that a set of most salient descriptions can be utilized. For large-scale models, feature selection is of utter importance in computation reduction and efficiency improvement. 

The distance evaluation technique ranks the feature importance independent of the model used for classification, which belongs to the filter category as mentioned in the Introduction. Salient features result in minimum inner-class distances of the same class while have maximum margins for different classes. It has been widely used in fault diagnosis of rotating machinery [20,21,38]. Suppose a feature set has *K* conditions, $\left\{q_{i,k,j},\;i=1,2,\ldots ,I_{k};\;k=1,2,\ldots ,K;\;j=1,2,\ldots ,J\right\}$, where $q_{i,k,j}$ is the *j*th eigenvalue of the *i*th sample under the *k*th condition, $I_{k}$ is the sample number of the *k*th condition, and *J* is the feature number of each sample. Totally $I_{k}\times K\times J$ features are obtained in the feature set $\left\{q_{i,k,j}\right\}$. Herein, a modified distance evaluation algorithm is presented as follows:

(1) Calculate the average distance of the same condition samples:
  (8) $$d_{k,j}=\frac{1}{I_{k}\times (I_{k}-1)}\sum_{l,i=1}^{I_{k}}\left|q_{i,k,j}-q_{l,k,j}\right|,\;l,i=1,2,\ldots ,I_{k},\;l\neq i$$
  then obtain the average distance of *K* conditions:
  (9) $$d_{j}^{\left(w\right)}=\frac{1}{K}\sum_{k=1}^{K}d_{k,j}$$
(2) Calculate the average eigenvalue of all samples under the same condition:
  (10) $$u_{k,j}=\frac{1}{I_{k}}\sum_{i=1}^{I_{k}}q_{i,k,j}$$
  then obtain the average distance between condition samples:
  (11) $$d_{j}^{\left(b\right)}=\frac{1}{K(K-1)}\sum_{k,e=1}^{K}\left|u_{e,j}-u_{k,j}\right|,\;k,e=1,2,\ldots ,K,\;k\neq e$$
(3) Calculate the variance factor of $d_{j}^{\left(b\right)}$ as:
  (12) $$v_{j}^{\left(b\right)}=\frac{\mathrm{sum}(\left|u_{e,j}-u_{k,j}\right|)}{\min(\left|u_{e,j}-u_{k,j}\right|)}$$
(4) Calculate the compensation factor as:
  (13) $$\delta_{j}=\frac{\mathrm{sum}(v_{j}^{b})}{v_{j}^{\left(b\right)}}$$
(5) Calculate the ratio $d_{j}^{\left(b\right)}$ and $d_{j}^{\left(w\right)}$ considering the compensation factor:
  (14) $$\alpha_{j}=\delta_{j}\frac{d_{j}^{\left(b\right)}}{d_{j}^{\left(w\right)}}$$
  then normalize $\alpha_{j}$ and obtaining the feature importance criteria:
  (15) $${\bar{\alpha }}_{j}=\frac{\alpha_{j}}{sum(\alpha_{j})}$$

A higher ${\bar{\alpha }}_{j}$ indicates that the corresponding feature *j* has greater importance. Features can be ranked in terms of the ${\bar{\alpha }}_{j}$ values in Equation (15) in descending order. This algorithm is referred to as Modified Distance Evaluation algorithm (MDE). Although the top ranked features have superior discriminative capability, they may suffer from high multi-collinearity, which refers to the non-independence among features [39]. Herein, the variance inflation factor (VIF) is used to avoid high collinearity. Assuming a training sample set *X* with *J* features $X_{1},X_{2},\ldots ,X_{J}$ and class *Y*, the VIF of feature *j* is calculated as:

(16) $${\mathrm{VIF}}_{j}=\frac{1}{1-R_{j}^{2}}$$

where $R_{j}^{2}$ is the R-squared value of the regression equation $X_{j}=\beta_{0}+\beta X^{\prime }$, in which $X^{\prime }$ contains all features except $X_{j}$. An improved algorithm combining MDE and VIF is presented in Algorithm 1 and is abbreviated as MDV (Modified Distance evaluation and variance inflation Factor).

**Algorithm 1:** MDV Algorithm.
(1)Set the selected future subset F_sub_ = ∅, *j* = 1;(2)Rank the *J* features in terms of the ${\bar{\alpha }}_{j}$ defined in Equation (15) in descending order. Set F_r_ to represent the index list of the ranked features. Add the first feature in F_r_ to F_sub_, *j* = *j* + 1;(3)while *j* < *J* :calculate the VIF_j_ of the *j*th feature in F_r_ with the features in F_sub_;if VIF_j_ < 10:     add the *j*th feature in F_r_ to F_sub_;end*j* = *j* + 1;end


The MDV algorithm describes the feature-subset selection for multi-class classification based on the filter method with the MDE and VIF. The threshold of 10 in MDV is an empirical value. A larger threshold will result in a higher correlation of the selected feature in F_r_ with the existing features in F_sub_ [23].

## 4. Case Study

### 4.1. Data Prepraration

A series of wind tunnel experiments of the self-sensing composite wing were conducted under various angles of attack (AoAs) and freestream velocities at Stanford University. The open-loop wind tunnel with a square test section of 0.76 m by 76 m was used and a basis was designed to supported the composite wing allowing adjustments in the angle of attack (AoA). The composite wing dimension is outlined in Table 4. 

Compared with the size of the wind tunnel test section, the additional 0.1 m extension of the wing span was attached to the wing fixture. The AoAs range from 0 degree up to 18 degrees with an incremental step of 1 degree. At each degree, data were collected for all velocities ranging from 9 to 22 m/s (incremental step of 1 m/s). For experimental details, please refer to [2]. 

PZT signals reflect the coupled airflow-structural dynamics through the wing structural vibration and each time series contains coupled behavior with repeated patterns of a certain flight state. This study focuses on the usage of PZT sensor signals for flight state identification. In each experiment, the structural vibration responses (60,000 data points) were recorded from the PZT located near the wing root at 1000 Hz sampling frequency. For each flight state, data are prepared in two steps: (1) the entire signal of 60,000 data points is divided into 60 segments (1000 data points for each segment) to ensure enough samples for training while each segment has sufficient data points for feature extraction; (2) the first order difference and zero-mean are conducted for each sample sequence in order to eliminate the influence of zero drift. To evaluate the effectiveness of the proposed method and apply it for dangerous state pre-warning, two sets of data are collected for general flight state identification and stall detection and alerting. 

### 4.2. General Flight State Identification

**Figure 3:**
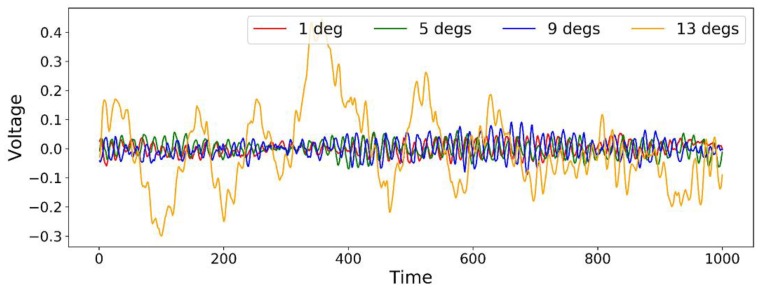
Indicative signals under a set of AoAs and a constant velocity of 10 m/s.

The first data set includes PZT signals with a coarse resolution covering the range of 16 flight states corresponding to combinations of four AoAs (1, 5, 9, 13 degrees) and four airspeeds (10, 13, 16, 19 m/s). Four signal segments are shown in Figure 3 under a series of AoAs and a fixed airspeed of 10 m/s as an example.

It is noticed that the flight state with AoA of 13 degrees and velocity of 10 m/s can be obviously identified since the amplitude of the voltage distinguishes it from other signals (it is because this flight state is close to the stall condition which will be discussed later). The second largest amplitude comes with 9 degrees which can be separated to a certain extent but already has overlaps with the rest two. In the study, the identification of the different flight states relies on the features selected by the developed method in Section 3. To compare the feature selection effectiveness, four other feature selection methods are employed including Univariate Feature Selection based on mutual information (UFS_m), Support Vector Machine with L1 regularization (SVM_L1), Gradient Boosted Decision Tree (GBDT) and Stability selection (STAB). These methods cover three main feature selection categories. A brief introduction is presented as follows:

(1) *UFS_m* is a commonly used filter method. It performs test on each feature by evaluating the relationship between the feature and the response variable based on mutual information [40], which is defined as
  (17) $$I(X,Y)=\sum_{y\in Y}\sum_{x\in X}p(x,y)\log(\frac{p(x,y)}{p(x)p(y)})$$
  It measures the mutual dependence between variable *X* and *Y*. Features with low rankings are removed.
(2) *SVM_L1* is one of the embedded methods, which selects salient features as part of the learning system [18]. Support Vector Machine (SVM) is a popular machine learning method based on structural risk minimization principle. It constructs a hyperplane that has the largest distance to the nearest training data points, which are so called support vectors. An appropriate separation can reduce the generalization error of the classifier [41]. L1 is a regularization item added to the loss function as |W|, where W standards for the parameter matrix of the learning model [42]. This is a penalty item to make the model sparse with fewer useful input dimensions.
(3) *GBDT* is a tree-based model belonging to the embedded category. It combines weak decision trees in an iterative manner based on gradient descent through additive training. Trees are added at each iteration with modified parameters learned in the direction of residual loss reduction [43].
(4) *Stability selection* is a kind of wrapper method, in which features are selected based on the established models using different subsets, model could be of various types and structures such as logistic regression, SVM, etc. By calculating the frequency of a feature ended up being selected as important from a feature subset being tested, powerful features are expected to have high scores close to 100%, weaker features will have lower score and the least useful ones will close to zero [44]. Herein, a randomized logistic regression is used as the selection model.

### 4.3. Application to Stall Detection and Identification

**Figure 4:**
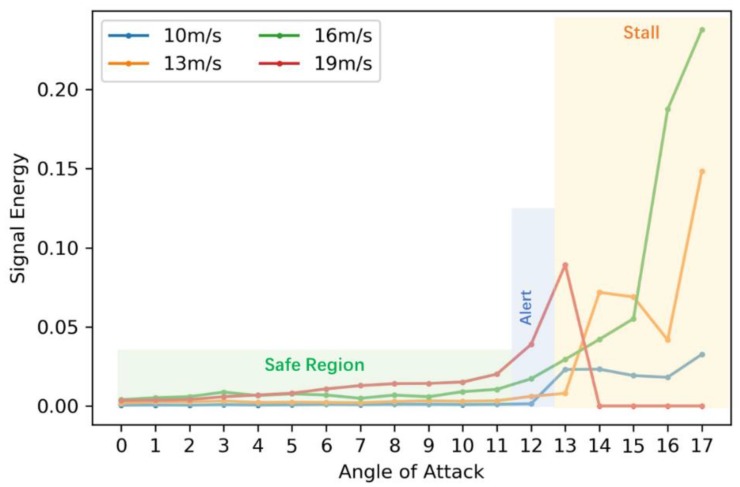
Signal energy under various flight states.

The second data set covers a higher resolution of flight states (AoAs: 11, 12, 13 degrees, airspeeds: 10, 13, 16, 19 m/s) for critical states alerting. In aerodynamics, stall phenomenon is one of the dangerous conditions wherein a sudden reduction of the lift coefficient occurs as the angle of attack increases beyond a critical point. According to previous analysis [2], the signal energy can be used as an indicator of the lift loss of the self-sensing wing. From the wind tunnel experiments, the mean values of the signal energy for a series of AoAs (from 0 to 17 degrees) under four airspeeds (10, 13, 16, 19 m/s) are obtained and shown in Figure 4.

The signal energy variation with respect to the angle of attack is similar under four different airspeeds. It is noticed that for relatively low velocities (10 m/s, 13 m/s & 16 m/s), the significant increase occurs approximately after 14 degrees while for the relatively high speed (19 m/s), stall happens much early at 13 degrees. It should be noted that the data were stopped recording after 13 degrees with the high speed of 19 m/s, which is reflected in the red line with zero energy starting from 14 degrees. Therefore, we define the orange shaded area starting from 13 degrees as the stall region which should be avoided. Moreover, it is observed that at 12 degrees, the signal energy for some flight states has certain increase compared with the rest small angles. This degree is defined as the alert region as the transition between the safe region marked in light green and the critical stall region. When the self-sensing wing comes to this region, warnings should be provided to the flight control for angle reduction. 

## 5. Results and Discussion

### 5.1. General Flight State Identification

The first data set with a relatively low resolution of 16 flight states is used to evaluate the performance of six feature selection methods, which include Univariate Feature Selection based on mutual information (UFS_m), Support Vector Machine with L1 regularization (SVM_L1), Gradient Boosted Decision Tree (GBDT) and Stability selection (STAB), Modified Distance Evaluation (MDE), and our proposed filter method Modified Distance Evaluation with Variance Inflation Factor (MDV). Feature rankings are obtained and the top 10 features for different methods are listed in Table 5 and their detailed expressions are listed in Appendix A. 

**Figure 5:**
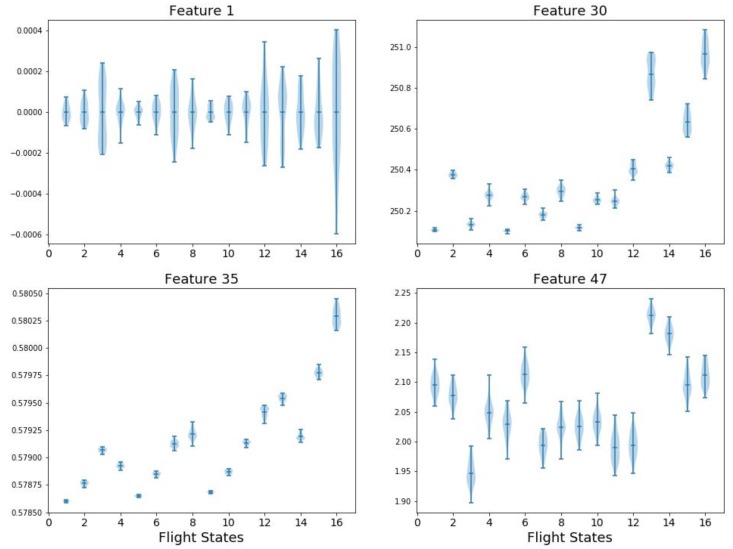
Pool and superior features against 16 flight states.

It is observed from the table that the ranking results vary with the different methods. An intuitive evaluation is to simply visualize the features distribution under various flight states. For example, four features are plotted in Figure 5 including: F1 (mean value in time domain), F29 (spectrum kurtosis in frequency domain), F35 (spectrum power convergence in frequency domain), and F47 (Hurst Exponent in information domain). The *x* axis denotes the 16 flight states while the *y* axis is the feature value before normalization. The shaded area along each vertical line segment represents the feature distribution in a single flight state and each subplot of Figure 5 describes a feature distribution on 16 flight states. As mentioned in Section 3, F1 (mean value) has no effects in classification. Correspondingly, F1 has the highest overlap among flight states. Similarly, F47 has large overlaps which exhibits pool classification capability. Theoretically, the ranking of F1 and F47 should be low but they are ranked high in GBDT and STAB. In comparison, F30 and F35 show smaller overlap and thus have better classification performance. This may provide some physical insights of the effectiveness of different feature selection methods.

**Figure 6:**
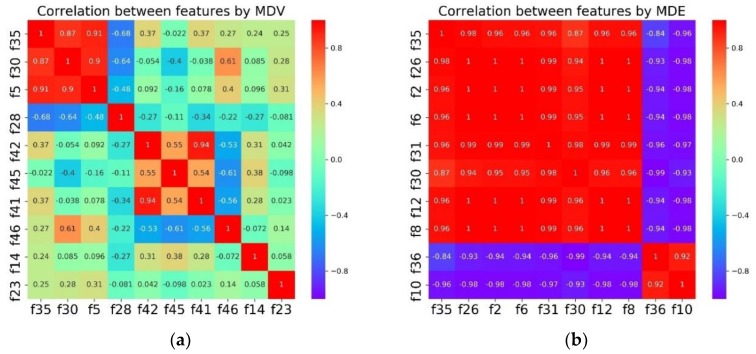
Correlation between features by MDV (**a**) and MDE (**b**).

The last column MDV in Table 4 is an improvement of MDE for preventing high collinearity. To examine the effects of the proposed algorithm, Correlation analysis is conducted for MDV and MDE as shown in Figure 6.

It is obvious that the top 10 features selected by MDE are highly correlated with each other. In comparison, the overall collinearity of the features in MDV is much lower except for the small region of the top three.

**Figure 7:**
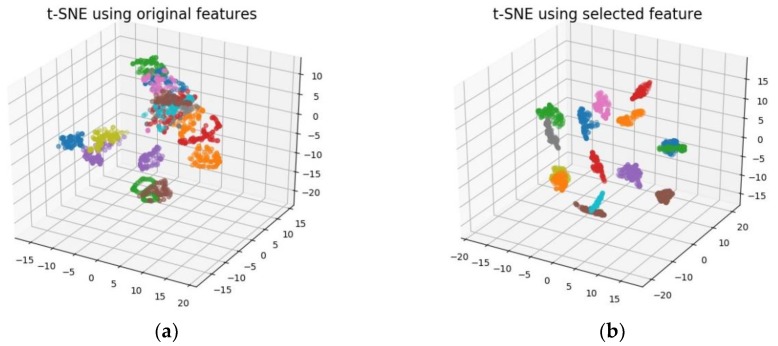
3D visualization by t-SNE: (**a**) t-SNE using original features; (**b**) t-SNE using selected features.

To visualize the feature selection performance by MDV, t-Distributed Stochastic Neighbor Embedding (t-SNE) is employed which is a relatively new method of dimension reduction particularly suitable for non-linear and high-dimensional datasets. It is a kind of manifold learning technique by mapping to probability distributions through affine transformation. For detailed algorithm, please refer to [45]. The 3D visualization by t-SNE is shown in Figure 7. The left figure is the visualization using the entire feature pool while the right figure uses only top six features obtained by MDV. It can be seen that the feature subset through MDV selection exhibits better classification effects compared to the entire feature pool.

**Figure 8:**
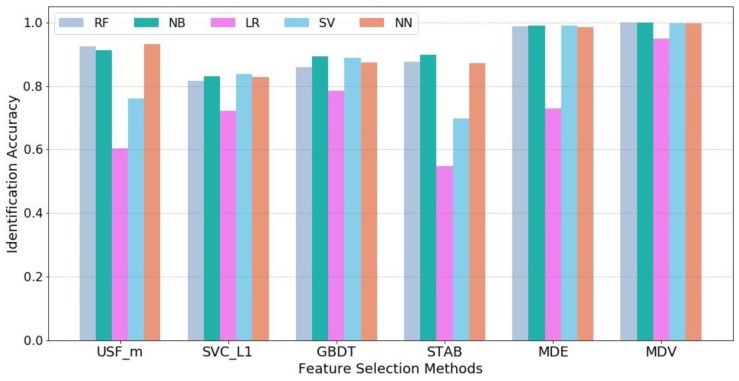
Identification accuracy against different feature selection methods.

Further, machine learning techniques are used to quantify the flight state identification process. For each feature selection method, the most salient 6 features are obtained as model inputs and the 16 flight states are set as model outputs. Five supervised learning models are employed including Logistic Regression (LR), Support Vector Machine (SVM), Naïve Bayes (NB), Random Forest (RF), and Neural Network (NN). Cross-validation is used in each model and the average accuracy value of five tests is computed to reduce the unbalance influence between training and testing samples. It should be noted that since the objective of the case study is to compare the effects of different feature selection methods instead of obtaining the optimized parameter setting for each machine learning model to achieve the highest accuracy level, default parameter settings in Python scikit-learn package for LR, SVM, NB and RF are used and remain the same for all feature selection methods while for NN, the parameter setting is as follows: {hidden layer size = 20, solver = ‘lbfgs’, activation function = ’relu’, learning rate = 0.001, maximum iteration = 100}. The identification results are shown in Figure 8.

It can be observed that our proposed method MDV achieves the highest identification accuracy in all five machine learning models and particularly, there is a significant improvement in Logistic Regression. This demonstrates the superior effectiveness of MDV. The comparison between MDV and MDE shows that a group of individually powerful features with low collinearity can lead to better results. 

### 5.2. Stall Detection and Alerting

So far, the developed MDV algorithm has achieved the best performance in feature selection and the final flight state identification accuracy is up to 100%. Herein, the second dataset with higher resolution is used for the application of stall detection and alerting. Similarly, totally 47 features as discussed in Section 3 are extracted and the most salient 6 features are selected by MDV as model inputs. A neural network is employed with the same parameter settings as the first case. The split rule is 80% samples for training and 20% samples for testing. 

The classification report is shown in Table 6 including three criteria: Precision, Recall and F1-score. Precision is the ratio of correctly predicted positive observations to the total predicted positive observations while Recall is the ratio of correctly predicted positive observations to the all observations in the actual class. F1-Score is the weighted average of Precision and Recall: F1-Score = 2 * (Recall * Precision)/(Recall + Precision) [46]. Safe, Alert, and Stall regions are divided with corresponding flight states. The overall identification accuracy is 98%.

**Figure 9:**
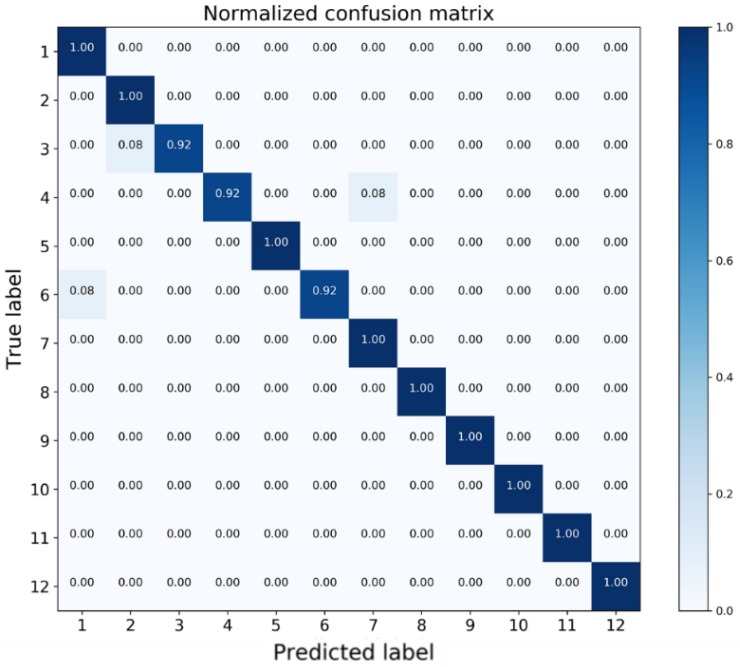
Confusion matrix of flight state identification.

To facilitate detailed analysis, a normalized confusion matrix is presented in Figure 9. Each row of the matrix represents the test samples in a true class label while each column indicates the samples in a predicted class label [47]. As can be observed from Table 6, for stall states (ID: 9, 10, 11, 12), Recall values all equal to 100%, meaning that all the critical states can be successfully identified and there is no safety risk.

In terms of alert states (ID: 5, 6, 7, 8), Recall value of State 6 is 0.92, which means 92% samples in State 6 are correctly predicted. By examining the 6th row in the confusion matrix, the rest 8% samples are misclassified as State 1, which is in the safe region. This situation may lead to dangerous results since the wing is already in the alert states yet there is no warning. From the other perspective, the precision value of State 7 is 0.92, which indicates that among all samples predicted as State 7, there are 8% samples actually belonging to State 4 as shown in the 7th column of the confusion matrix. This value can be interpreted as the false-alarm ratio that the wing flying in the safe region yet receives a false alert. 

For safe states (ID: 1, 2, 3, 4), the misclassified samples are for State 3 and State 4, in which 8% samples of State 3 are predicted as State 2 while 8% samples of State 4 are identified as State 7, which is the false alarm. 

**Figure 10:**
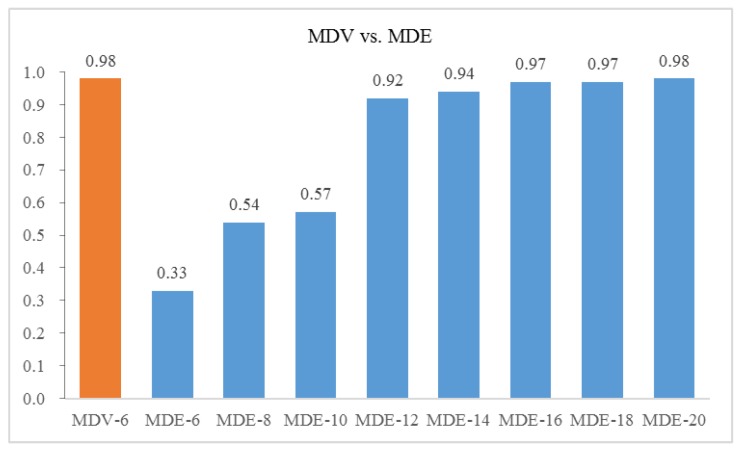
Identification accuracy between MDV and various MDE.

Further, we select the different number of features from the modified distance evaluation (MDE) method and use the same neural network structure for training and testing. The comparison on the overall identification accuracy between MDV and various MDE is shown in Figure 10. The *x* axis denotes number of top ranked features selected.

It can be seen that if we use the same number of input as MDV, features selected by MDE lead to a pool result of 0.33. The identification accuracy reaches the same level as MDV until the number of top ranked features selected from MDE increases to 20. This shows that our proposed method MDV is able to address the collinearity problem and uses fewer features to achieve superior performance with a considerable model complexity reduction.

## 6. Conclusions

This paper focuses on the feature engineering in structural vibration signals obtained from a self-sensing composite wing through wind tunnel experiments. In addition to common statistical features from the time domain and frequency domain, complex features from the information domain inspired by electroencephalograph analysis and mechanical fault diagnosis are also extracted, some of which exhibit good classification ability. A novel filter feature selection method (MDV) is proposed by combining the modified distance evaluation (MDE) algorithm and the variance inflation factor (VIF). MDE is able to select individually powerful features but cannot address high collinearity. VIF is then applied for each top ranked feature to remove highly correlated elements. Results from both general flight state identification and stall detection & alerting demonstrate that this method can reduce the model complexity with fewer features while maintain a high identification accuracy. Knowledge can be gained by calculating the limited important features obtained by MDV efficiently for flight state identification using light-weight machine learning models. This would save considerable efforts in feature extraction and feature selection by manpower and has the potential to provide autonomous control with real-time flight state monitoring. For multi-sensor utilizations, this method can be applied to each sensor and ensemble methods can be developed to fuse multi-source results for more robust identification.

## Figures and Tables

**Table 1:** Features in time domain.

Time Domain Feature Parameters		Un-Dimensional	
$t_{1}=\frac{\sum_{n=1}^{N}x(n)}{N}$	$t_{7}={\left(\frac{\sum_{n=1}^{N}\sqrt{\left|x(n)\right|}}{N}\right)}^{2}$	$t_{13}=\frac{t_{9}}{t_{6}}$	$t_{19}=\frac{\sum_{n=1}^{N}{\left(x(n)-t_{1}\right)}^{3}}{N\cdot t_{2}{}^{3}}$
$t_{2}=\sqrt{\frac{\sum_{n=1}^{N}{\left(x(n)-t_{1}\right)}^{2}}{N}}$	$t_{8}=\frac{\sum_{n=1}^{N}\left|x(n)\right|}{N}$	$t_{14}=\frac{t_{6}}{t_{8}}$	$t_{20}=\frac{\sum_{n=1}^{N}{\left(x(n)-t_{1}\right)}^{4}}{N\cdot t_{2}{}^{4}}$
$t_{3}=\frac{\sum_{n=1}^{N}{\left(x(n)-t_{1}\right)}^{3}}{N}$	$t_{9}=\max(x(n))$	$t_{15}=\frac{t_{9}}{t_{8}}$	…
$t_{4}=\frac{\sum_{n=1}^{N}{\left(x(n)-t_{1}\right)}^{4}}{N}$	$t_{10}=\min(x(n))$	$t_{16}=\frac{t_{9}}{t_{7}}$	
$t_{5}=\frac{\sum_{n=1}^{N}{\left(x(n)-t_{1}\right)}^{2}}{N}$	$t_{11}=t_{9}-t_{10}$	$t_{17}=\frac{t_{3}}{t_{6}{}^{3}}$	
$t_{6}=\sqrt{\frac{\sum_{n=1}^{N}{\left(x(n)\right)}^{2}}{N}}$	$t_{12}=\frac{\sum_{n=1}^{N}\left|x(n)-t_{1}\right|}{N}$	$t_{18}=\frac{t_{4}}{t_{6}{}^{4}}$	$t_{25}=\frac{\sum_{n=1}^{N}{\left(x(n)-t_{1}\right)}^{9}}{N\cdot t_{2}{}^{9}}$

Note: *x*(*n*) is a signal series for n = 1, 2, …, *N*, *N* is the number of data points.

**Table 2:** Features in the frequency domain.

Frequency Domain Feature Parameters		
$f_{1}=\frac{\sum_{k=1}^{K}y(k)}{N}$	$f_{6}=\sqrt{\frac{\sum_{k=1}^{K}{\left(fr_{k}-f_{5}\right)}^{2}y(k)}{K}}$	$f_{10}=\frac{f_{6}}{f_{5}}$
$f_{2}=\frac{\sum_{k=1}^{K}{\left(y(k)-f_{1}\right)}^{2}}{K}$	$f_{7}=\sqrt{\frac{\sum_{k=1}^{K}fr_{k}{}^{2}y(k)}{\sum_{k=1}^{K}y(k)}}$	$f_{11}=\frac{\sum_{k=1}^{K}{\left(fr_{k}-f_{5}\right)}^{3}y(k)}{K\cdot f_{6}{}^{3}}$
$f_{3}=\frac{\sum_{k=1}^{K}{\left(y(k)-f_{1}\right)}^{3}}{K{\left(\sqrt{f_{2}}\right)}^{3}}$	$f_{8}=\sqrt{\frac{\sum_{k=1}^{K}fr_{k}{}^{4}y(k)}{\sum_{k=1}^{K}fr_{k}{}^{2}y(k)}}$	$f_{12}=\frac{\sum_{k=1}^{K}{\left(fr_{k}-f_{5}\right)}^{4}y(k)}{K\cdot f_{6}{}^{4}}$
$f_{4}=\frac{\sum_{k=1}^{K}{\left(y(k)-f_{1}\right)}^{4}}{K\cdot f_{2}{}^{2}}$	$f_{9}=\frac{\sum_{k=1}^{K}fr_{k}{}^{2}y(k)}{\sqrt{\sum_{k=1}^{K}y(k)\sum_{k=1}^{K}fr_{k}{}^{4}y(k)}}$	$f_{13}=\frac{\sum_{k=1}^{K}\sqrt{\left|fr_{k}-f_{5}\right|}y(k)}{K\sqrt{f_{6}}}$
$f_{5}=\frac{\sum_{k=1}^{K}\left(fr_{k}\cdot y(k)\right)}{\sum_{k=1}^{K}y(k)}$		

Note: *y*(*k*) is a spectrum for *k =* 1, 2, *…*, *K*, *K* is the number of spectrum components;$fr_{k}$ is the frequency value of the *k*th spectrum line.

**Table 3:** Features in information domain.

Information Domain Feature Parameters		
I_1_ = MSE [1]	I_4_ = PMMSE	I_7_ = FI
I_2_ = MSE [2]	I_5_ = PFD	I_8_ = ApEn
I_3_ = MSE [3]	I_6_ = HFD	I_9_ = HST

**Table 4:** Wing Dimension.

Wing Geometry	
Chord	0.235 m
Span	0.86 m
Area	0.2 m^2^
Aspect ratio	3.66

**Table 5:** Top 10 ranking matrix.

Ranking	UFS_m	SVM_L1	GBDT	STAB	MDE	MDV
1	F25	F41	F47	F47	F35	F35
2	F34	F43	F40	F12	F26	F30
3	F6	F39	F46	F21	F2	F5
4	F2	F25	F14	F20	F6	F28
5	F5	F46	F39	F19	F31	F42
6	F4	F19	F44	F18	F30	F45
7	F40	F33	F41	F17	F12	F41
8	F23	F13	F1	F16	F8	F46
9	F42	F44	F21	F15	F36	F14
10	F17	F10	F45	F14	F10	F23

**Table 6:** Classification report.

	States ID	AoA (deg)	Speed (m/s)	Precision	Recall	F1-Score
**Safe**	1	11	10	0.92	1.00	0.96
	2	11	13	0.92	1.00	0.96
	3	11	16	1.00	0.92	0.96
	4	11	19	1.00	0.92	0.96
**Alert**	5	12	10	1.00	1.00	1.00
	6	12	13	1.00	0.92	0.96
	7	12	16	0.92	1.00	0.96
	8	12	19	1.00	1.00	1.00
**Stall**	9	13	10	1.00	1.00	1.00
	10	13	13	1.00	1.00	1.00
	11	13	16	1.00	1.00	1.00
	12	13	19	1.00	1.00	1.00

## Appendix A

The expressions of selected features by different feature selection methods are shown in Table A1.

**Table A1:** Top 10 ranking feature expressions.

R	UFS_m	SVM_L1	GBDT	STAB	MDE	MDV
1	$t_{25}=\frac{\sum_{n=1}^{N}{\left(x(n)-t_{1}\right)}^{9}}{N\cdot t_{2}{}^{9}}$	I_3_ = MSE[3]	I_9_ = HST	I_9_ = HST	$f_{10}=\frac{f_{6}}{f_{5}}$	$f_{10}=\frac{f_{6}}{f_{5}}$
2	$f_{9}=\frac{\sum_{k=1}^{K}fr_{k}{}^{2}y(k)}{\sqrt{\sum_{k=1}^{K}y(k)\sum_{k=1}^{K}fr_{k}{}^{4}y(k)}}$	I_5_ = PFD	I_2_ = MSE[2]	$t_{12}=\frac{\sum_{n=1}^{N}\left|x(n)-t_{1}\right|}{N}$	$f_{1}=\frac{\sum_{k=1}^{K}y(k)}{N}$	$f_{5}=\frac{\sum_{k=1}^{K}\left(fr_{k}\cdot y(k)\right)}{\sum_{k=1}^{K}y(k)}$
3	$t_{6}=\sqrt{\frac{\sum_{n=1}^{N}{\left(x(n)\right)}^{2}}{N}}$	I_1_ = MSE[1]	I_8_ = ApEn	$t_{21}=\frac{\sum_{n=1}^{N}{\left(x(n)-t_{1}\right)}^{5}}{N\cdot t_{2}{}^{5}}$	$t_{2}=\sqrt{\frac{\sum_{n=1}^{N}{\left(x(n)-t_{1}\right)}^{2}}{N}}$	$t_{5}=\frac{\sum_{n=1}^{N}{\left(x(n)-t_{1}\right)}^{2}}{N}$
4	$t_{2}=\sqrt{\frac{\sum_{n=1}^{N}{\left(x(n)-t_{1}\right)}^{2}}{N}}$	$t_{25}=\frac{\sum_{n=1}^{N}{\left(x(n)-t_{1}\right)}^{9}}{N\cdot t_{2}{}^{9}}$	$t_{14}=\frac{t_{6}}{t_{8}}$	$t_{20}=\frac{\sum_{n=1}^{N}{\left(x(n)-t_{1}\right)}^{4}}{N\cdot t_{2}{}^{4}}$	$t_{6}=\sqrt{\frac{\sum_{n=1}^{N}{\left(x(n)\right)}^{2}}{N}}$	$f_{3}=\frac{\sum_{k=1}^{K}{\left(y(k)-f_{1}\right)}^{3}}{K{\left(\sqrt{f_{2}}\right)}^{3}}$
5	$t_{5}=\frac{\sum_{n=1}^{N}{\left(x(n)-t_{1}\right)}^{2}}{N}$	I_8_ = ApEn	I_1_ = MSE[1]	$t_{19}=\frac{\sum_{n=1}^{N}{\left(x(n)-t_{1}\right)}^{3}}{N\cdot t_{2}{}^{3}}$	$f_{6}=\sqrt{\frac{\sum_{k=1}^{K}{\left(fr_{k}-f_{5}\right)}^{2}y(k)}{K}}$	I_4_ = PMMSE
6	$t_{4}=\frac{\sum_{n=1}^{N}{\left(x(n)-t_{1}\right)}^{4}}{N}$	$t_{19}=\frac{\sum_{n=1}^{N}{\left(x(n)-t_{1}\right)}^{3}}{N\cdot t_{2}{}^{3}}$	I_6_ = HFD	$t_{18}=\frac{t_{4}}{t_{6}{}^{4}}$	$f_{3}=\frac{\sum_{k=1}^{K}{\left(y(k)-f_{1}\right)}^{3}}{K{\left(\sqrt{f_{2}}\right)}^{3}}$	I_7_ = FI
7	I_2_ = MSE[2]	$f_{8}=\sqrt{\frac{\sum_{k=1}^{K}fr_{k}{}^{4}y(k)}{\sum_{k=1}^{K}fr_{k}{}^{2}y(k)}}$	I_3_ = MSE[3]	$t_{17}=\frac{t_{3}}{t_{6}{}^{3}}$	$t_{12}=\frac{\sum_{n=1}^{N}\left|x(n)-t_{1}\right|}{N}$	I_3_ = MSE[3]
8	$t_{23}=\frac{\sum_{n=1}^{N}{\left(x(n)-t_{1}\right)}^{7}}{N\cdot t_{2}{}^{7}}$	$t_{13}=\frac{t_{9}}{t_{6}}$	$t_{1}=\frac{\sum_{n=1}^{N}x(n)}{N}$	$t_{16}=\frac{t_{9}}{t_{7}}$	$t_{8}=\frac{\sum_{n=1}^{N}\left|x(n)\right|}{N}$	I_8_ = ApEn
9	I_4_ = PMMSE	I_6_ = HFD	$t_{21}=\frac{\sum_{n=1}^{N}{\left(x(n)-t_{1}\right)}^{5}}{N\cdot t_{2}{}^{5}}$	$t_{15}=\frac{t_{9}}{t_{8}}$	$f_{11}=\frac{\sum_{k=1}^{K}{\left(fr_{k}-f_{5}\right)}^{3}y(k)}{K\cdot f_{6}{}^{3}}$	$t_{14}=\frac{t_{6}}{t_{8}}$
10	$t_{17}=\frac{t_{3}}{t_{6}{}^{3}}$	$t_{10}=\min(x(n))$	I_7_ = FI	$t_{14}=\frac{t_{6}}{t_{8}}$	$t_{10}=\min(x(n))$	$t_{23}=\frac{\sum_{n=1}^{N}{\left(x(n)-t_{1}\right)}^{7}}{N\cdot t_{2}{}^{7}}$
